# Outer Membrane Vesicles (OMV)-based and Proteomics-driven Antigen Selection Identifies Novel Factors Contributing to *Bordetella pertussis* Adhesion to Epithelial Cells*[Fn FN2]

**DOI:** 10.1074/mcp.RA117.000045

**Published:** 2017-12-04

**Authors:** Gianmarco Gasperini, Massimiliano Biagini, Vanessa Arato, Claudia Gianfaldoni, Alessandro Vadi, Nathalie Norais, Giuliano Bensi, Isabel Delany, Mariagrazia Pizza, Beatrice Aricò, Rosanna Leuzzi

**Affiliations:** From the ‡GSK Vaccines, Siena, Italy

## Abstract

Despite high vaccination coverage world-wide, whooping cough, a highly contagious disease caused by *Bordetella pertussis,* is recently increasing in occurrence suggesting that novel vaccine formulations targeted at the prevention of colonization and transmission should be investigated. To identify new candidates for inclusion in the acellular formulation, we used spontaneously released outer membrane vesicles (OMV)^1^ as a potential source of key adhesins. The enrichment of Bvg+ OMV with adhesins and the ability of anti-OMV serum to inhibit the adhesion of *B. pertussis* to lung epithelial cells *in vitro* were demonstrated. We employed a proteomic approach to identify the differentially expressed proteins in OMV purified from bacteria in the Bvg+ and Bvg− virulence phases, thus comparing the outer membrane protein pattern of this pathogen in its virulent or avirulent state. Six of the most abundant outer membrane proteins were selected as candidates to be evaluated for their adhesive properties and vaccine potential. We generated *E. coli* strains singularly expressing the selected proteins and assessed their ability to adhere to lung epithelial cells *in vitro*. Four out of the selected proteins conferred adhesive ability to *E. coli*. Three of the candidates were specifically detected by anti-OMV mouse serum suggesting that these proteins are immunogenic antigens able to elicit an antibody response when displayed on the OMV. Anti-OMV serum was able to inhibit only BrkA-expressing *E. coli* adhesion to lung epithelial cells. Finally, stand-alone immunization of mice with recombinant BrkA resulted in significant protection against infection of the lower respiratory tract after challenge with *B. pertussis*. Taken together, these data support the inclusion of BrkA and possibly further adhesins to the current acellular pertussis vaccines to improve the impact of vaccination on the bacterial clearance.

*Bordetella pertussis* is a Gram-negative bacterium, obligate human pathogen and causative agent of whooping cough, a highly contagious disease which is recently increasing in occurrence despite high vaccination coverage world-wide (1–3). The resurgence of pertussis over the last two decades has been suggested to be because of many factors including improved diagnostics and pathogen evolution but also to waning immunity following vaccination with the acellular formulation (aP) which replaced the more reactogenic whole-cell vaccine (wP) (4–7).

Acellular pertussis vaccines are currently available from different manufacturers and include up to five different components (Pertussis Toxin (PT)), Filamentous Hemagglutinin (FHA), 69kDa outer-membrane protein (also known as Pertactin), fimbrial-2 and fimbrial-3 antigens) in different concentrations and with different adjuvants. All the aP vaccine components are highly regulated by the BvgAS two component system which enables *B. pertussis* to respond to extracellular stimuli and modulate the concerted activation of all the virulence genes acting like a master switch among clearly distinct phenotypic phases (8). Therefore, Bvg-activated proteins are mainly associated with colonization, toxicity and host immune evasion and represent potential vaccine candidates (9).

Importantly, several studies including the recent employment of the baboon infection model have shown that the acellular vaccine is able to prevent the clinical symptoms of the disease but not the colonization of the airways, leading to an increased risk of transmission and consequent bacterial spread throughout the population (10). Moreover, strains belonging to the *ptxP3* lineage have emerged in recent years, showing a higher level of PT and loss of Pertactin (11); therefore, aP vaccines may be less efficient in eliciting toxin-neutralizing and anti-adhesive antibodies against these new circulating strains. Taken together, all these data suggest that a new generation vaccine against pertussis able to shorten bacterial colonization by inclusion of new protective antigens is needed (12).

To identify new adhesins to be included in a novel vaccine formulation we used outer membrane vesicles (OMV) as a potential source for the identification of protective antigens. OMV are blebs of the outer membrane which are spontaneously released by all Gram-negative bacteria during growth and they contain periplasmic proteins in their lumen and outer membrane proteins and lipoproteins in their natural conformation and architectural context (13, 14).

In this study, we isolated OMV from the pathogen in its virulent (Bvg+) or avirulent (Bvg−) phase and employing a proteomic approach we selected six Bvg-regulated candidates to be subsequently evaluated for their adhesive properties and vaccine potential. Indeed, OMV are far more suitable than Outer Membrane Protein (OMP) preparations for proteomic analysis because of the lack of contaminants deriving from other cellular compartments such as the cytoplasm. Finally, we evaluated whether a stand-alone immunization with BrkA could confer protection in a mouse aerosol challenge model of infection.

## EXPERIMENTAL PROCEDURES

### 

#### 

##### Bacterial Strains and Growth Conditions

The following *B. pertussis* strains were used in this study: Tohama I-derivative BP536 (15) and BP537 (16) and W28 PT 9K/129G (17). Bacteria were stored at −80 °C and recovered by plating on Bordet-Gengou (BG) agar plates, supplemented with 15% (v/v) sheep blood, for 3 days at 37 °C. Bacteria were then inoculated at initial 600 nm optical density (OD_600_) of 0.05–0.1 in Stainer-Scholte medium supplemented with 0.4% (w/v) l-cysteine monohydrochloride, 0.1% (w/v) FeSO_4_, 0.2% (w/v) ascorbic acid, 0.04% (w/v) nicotinic acid, 1% (w/v) reduced glutathione. Cultures were grown in rotary shakers at 37 °C. Recombinant DH5α *E. coli* strains were stored at −80 °C, recovered by plating on LB agar plates supplemented with 20 μg/ml chloramphenicol for 16 h at 37 °C. For liquid cultures, bacteria were inoculated in LB medium supplemented with 20 μg/ml chloramphenicol and were grown in rotary shakers at 37 °C for 16 h.

##### Generation of E. coli Strains Expressing Heterologous B. pertussis Candidate Adhesins

*E. coli* strain DH5α was transformed with a range of 6 plasmids based on the broad host range vector pMMB208 (18) which was modified to express the candidate adhesins. The ApaI-XbaI fragment containing the *lacI* gene and the IPTG-inducible pTac promoter was substituted with an expressing cassette consisting of a constitutive *B. pertussis* promoter and the full-length coding sequences for *brkA*, *sphB1*, *vag8*, *tcfA*, *bipA* and *bfrD.* The promoter and the coding sequences were amplified from *B. pertussis* W28 9K/129G genomic DNA using the following primers: promoter F (ccc**GGGCCC**TCCTTGAGTGAACTGGGGG) and promoter R (ccc**AAGCTT**AATCTCCGTTGATTTGAGTGA); *brkA* F (ccc**AAGCTT**ATGTATCTCGATAGATTCCGTCAA) and *brkA* R (ccc**TCTAGA**TCAGAAGCTGTAGCGGTAGC); *sphB1* F (ccc**AAGCTT**GTGATGCCGCCGCCGGCCGT) and *sphB1* R (ccc**TCTAGA**TCAGTAGCGGTAAGTGAGGCT); *vag8* F (ccc**AAGCTT**ATGGCAGGACAAGCGAGG) and *vag8* R (ccc**TCTAGA**TCACCAGCTGTAGCGATACC); *tcfA* F (ccc**AAGCTT**ATGCACATTTACGGAAATATGAA) and *tcfA* R (ccc**TCTAGA**CTACCAGGCGTAGCGATAGC); *bipA* F (ccc**AAGCTT**ATGAACAAGAACATTTACCGTGTT) and *bipA* R (ccc**TCTAGA**TTAGTAAGGAAAATTGACCGGC); *bfrD* F (ccc**AAGCTT**ATGAAGTTCTACTCTTCCCATCC) and *bfrD* R (ccc**TCTAGA**TCAGTAGCTCAGCTTGAACGTC). The amplified promoter fragment was digested with ApaI/HindIII and the amplified CDS were digested with HindIII/XbaI and were ligated into the ApaI-XbaI digested vector and the final constructs were checked by sequencing. The plasmids were transformed into *E. coli* DH5α and stored at −80 °C.

##### OMV Purification

Cell-free supernatants from liquid cultures of BP536 and BP537 were recovered after a 3-day growth in 250 ml baffled flasks. The liquid-air volume ratio resulted critical for OMV production yield and was kept at 1:5 ratio. Bacteria were then pelleted through centrifugation at 5000 × *g* for 30 min. Supernatants were recovered and filtered through 0.22 μm Stericup filters (Millipore, Billerica, MA). After ultracentrifugation at 175,000 × *g* for 2 h at 4 °C, the resulting OMV pellet was washed with Dulbecco's Phosphate-Buffered Saline (d-PBS), further ultracentrifuged at 175,000 × *g* for 2 h and finally resuspended in 100 μl d-PBS. OMV were quantified through the Lowry assay (DC Protein Assay, BioRad, Hercules, CA) for total protein content following the manufacturer's instructions.

##### SDS-PAGE Analysis

For sodium dodecylsulfate-polyacrylamide gel electrophoresis (SDS-PAGE) analysis, 10 μg (protein content) of OMV samples were resuspended in 20 mm Tris-HCl (pH 8.0) buffer containing 8% (w/v) SDS and 10 mm DTT, boiled for 5 min, separated on NuPAGE™ Novex™ 4–12% polyacrylamide Bis-Tris Protein Gels (Invitrogen) and stained with Coomassie Blue R-250.

##### Nanoparticle Tracking Analysis

A NanoSight NS300 instrument (Malvern Ltd.) was used to determine OMV particle size and concentration as previously described (19). Briefly, OMV can be observed by light scattering using a light microscope. Sequential videos are recorded and the NTA software can track the Brownian movement of individual vesicles and calculate the size and concentration of OMV. Samples at the protein concentration of 1 mg/ml were diluted 1:500 or 1:1000 in d-PBS and loaded in the sample chamber. Five videos per samples were recorded for 60 s and size of individual OMVs and total amount of OMV particles were analyzed by Nanoparticle Tracking Analysis 3.2 software (NanoSight Ltd., Malvern, United Kingdom). All measurements were performed at room temperature.

##### Generation of Mouse Immune Sera

BALB/c mice (10 female/group, 6-week old) (Charles River Laboratories International Inc., Wilmington, MA) received three intraperitoneal immunizations, with a 4-week interval, with aluminum hydroxide adjuvanted OMV from *B. pertussis* strain W28 9K/129G (2.5 μg per dose) or wP vaccine (NIBSC) at one fifteenth of a human dose. Sera were collected before immunization and 2 weeks after the third immunization. Control mice immunized with adjuvant only were included in the experiments. All animal experiments were performed in compliance with the Italian law with the approval of the local Animal Welfare Body (AWB 2014/06) followed by authorization of Italian Ministry of Health.

##### Adhesion Assay

The A549 cell line (Human epithelial alveolar basal adenocarcinoma, ATCC CCL-185) was maintained in Ham's F-12K medium (Life Technologies) supplemented with 10% (v/v) heat-inactivated fetal bovine serum (FBS)(Gibco, Waltham, MA) and antibiotics. Cells were grown at 37 °C in humidified atmosphere containing 5% CO_2_. A549 cells were seeded on black 96-well plate (2.5 × 10^4^ cells/well) and cultured for 1 day in the absence of antibiotics.

For *B. pertussis* OMV adhesion assay, Bvg+ and Bvg− OMV were resuspended in d-PBS to the final concentration of 10 ng/μl (protein content) and 100 μl were transferred in triplicates on plated A549 cells. After 6 h of incubation, cells were washed extensively with d-PBS, then fixed with 3.7% (v/v) formaldehyde (Sigma) for 20 min, blocked with d-PBS containing 3% (w/v) Bovine Serum Albumin (BSA) (Sigma) for 15 min and incubated with mouse anti-OMV serum diluted in d-PBS with 1% (w/v) BSA (1:5000) for 1 h. After washes, samples were incubated with Alexa Fluor 488 rabbit anti-mouse IgG (1:500) (Molecular Probes) for 30 min. After three washes with d-PBS, fluorescence was measured at excitation/emission 485/535 nm by Tecan Infinite F200PRO microplate reader.

For *E. coli* adhesion assay, bacteria were grown for 16 h in liquid culture and then washed with d-PBS, centrifuged at 8,000 × *g* for 5 min and resuspended at OD_600_ 0.1 in F12-K medium. One hundred microliters of the bacterial suspension were transferred in triplicate onto plated A549 cells. Infected cells were incubated for 3 h at 37 °C. After extensive washing to remove unbound bacteria, cells were fixed with 3.7% (v/v) formaldehyde for 20 min, blocked with d-PBS containing 3% (w/v) BSA for 15 min and incubated with rabbit anti-*E. coli* polyclonal antibodies diluted in d-PBS with 1% (w/v) BSA (1:500) for 1 h. After washes, samples were incubated with Alexa Fluor 488 goat anti-rabbit IgG (1:500) (Molecular Probes, Eugene, OR) for 30 min. After three washes with d-PBS, fluorescence was measured at excitation/emission 485/535 nm by Tecan Infinite F200PRO microplate reader.

Adhesion assays were performed 3 times on different days; statistical analyses were performed using unpaired *t* test (for *B. pertussis* OMV adhesion) and one-way ANOVA with Dunnett's multiple comparison test (for *E. coli* adhesion).

##### Adhesion Inhibition Assay

For *B. pertussis* adhesion inhibition assay, bacteria were grown for 16 h in liquid culture and then pelleted at 8000 × *g* for 5 min and resuspended in d-PBS at OD_600_ 0.5. For the fluorescent labeling of *B. pertussis* cells, a volume of 445 μl of bacterial suspension was then mixed with 50 μl of 1 m NaHCO_3_ and 5 μl of Alexa Fluor 488 Carboxylic Acid, Succinimidyl Ester (Life Technologies, Waltham, MA) and incubated for 15 min at 37 °C. After centrifugation at 8,000 *x g* for 5 min at room temperature, supernatant was removed and pellet was washed once with 1 ml d-PBS to remove unbound dye and bacteria were finally resuspended in F12-K medium at OD_600_ 0.2. Pooled mouse sera were 4-fold serially diluted in F-12K medium containing 1% (v/v) naïve mouse serum and incubated with labeled *B. pertussis* for 1 h at 37 °C in 1:1 ratio. One hundred microliters of bacteria/serum mixtures were transferred in triplicate onto plated A549 cells. Infected cells were incubated for 1 h at 37 °C. After extensive washing to remove unbound bacteria, fluorescence was measured at excitation/emission 485/535 nm by Tecan Infinite F200PRO microplate reader.

For *E. coli* adhesion inhibition assay, we used the same protocol used for *B. pertussis* adhesion inhibition assay, but we revealed *E. coli* adhering bacteria by immuno-fluorescence using rabbit anti-*E. coli* polyclonal antibodies as previously described for the adhesion assay.

##### Proteomic Analysis by LC-MS/MS

For quantitative proteomic analysis, one hundred micrograms of OMV were TCA precipitated as previously described (20) and the protein pellet was resuspended in 50 mm ammonium bicarbonate containing 0.1% (w/v) RapiGest SF™ (Waters, Milford, MA) and 5 mm DTT and heated at 100 °C for 10 min. Digestions were performed overnight at 37 °C with 2.5 μg trypsin (Promega, Fitchburg, WI). Digestions were stopped with 0.1% (v/v) formic acid, desalted using OASIS HLB cartridges (Waters) as described by the manufacturer, dried in a Centrivap Concentrator (Labconco, Kansas City, MO) and resuspended in 50 μl of 3% (v/v) ACN and 0.1% (v/v) formic acid. Peptide mixtures were stored at −20 °C until further analysis. An Acquity UPLC instrument (Waters) was coupled on-line to a Q Exactive Plus (Thermo Fisher Scientific) with an electrospray ion source (Thermo Fisher Scientific). The peptide mixture (10 μg) was loaded onto a C18-reversed phase column Acquity UPLC peptide CSH C18 130Å, 1.7 μm 1 × 150 mm and separated with a linear gradient of 28–85% buffer B (0.1% (v/v) formic acid in ACN) at a flow rate of 50 μl/min and 50 °C. MS data was acquired in positive mode using a data-dependent acquisition (DDA) dynamically choosing the five most abundant precursor ions from the survey scan (300–1600 *m*/*z*) at 70,000 resolution for HCD fragmentation. Automatic Gain Control (AGC) was set at 3 × 10^6^. For MS/MS acquisition, the isolation of precursors was performed with a 3 *m*/*z* window and MS/MS scans were acquired at a resolution of 17,500 at 200 *m*/*z* with normalized collision energy of 26 eV.

##### Experimental Design and Statistical Rationale

For each of three biological replicates of OMV purified from Bvg+ and Bvg− strains, three LC-MS/MS acquisitions were performed (technical replicates).

Because the Bvg regulon is intrinsically very sensitive to little environmental changes (such as culture media composition and temperature) and given that we are not using controlled growth settings like fermentors, we decided to analyze each biological replicate independently.

The percentage of each protein in the total sample was calculated for each biological replicate according to the corresponding peak area (averaged among the three technical replicates) and the theoretical molecular weight (MW) using the following formula:

$$\%\text{ProteinX}=\frac{AvgAreaProteinX*MWProteinX}{\sum \left(AvgAreaProtein*MWProtein\right)}$$

Then, the fold change of each protein amount in Bvg+ *versus*, Bvg− was calculated. Finally, we set 2 parameters to define Bvg+ phase specificity: a protein is considered specific to Bvg+ OMV if its amount is at least 4-fold higher in Bvg+ *versus* Bvg− and if it represents less than 0.5% of the total amount of proteins in Bvg− OMV.

##### Proteomic Data Analysis

The mass spectrometric raw data were processed with the PEAKS software ver. 8 (Bioinformatics Solutions Inc., Waterloo, Ontario, Canada) for *de novo* sequencing, database matching identification and label free quantification. Raw mass spectrometry data were deposited in the publicly accessible repository MassIVE (Project number: MSV000081702; Proteome Exchange PXD008179). Peptide scoring for identification was based on a database search with an initial allowed mass deviation of the precursor ion of up to 15 ppm. The allowed fragment mass deviation was 0.05 Da. Protein identification from MS/MS spectra was performed against *B. pertussis* Tohama I NCBI protein database (3,425 protein entries, release date: November 6, 2001) combined with common contaminants (human keratins and autoproteolytic fragments of trypsin) with a False discovery rate (FDR) set at 0.1%. FDR is defined as the ratio between the false peptide-spectrum match (PSMs) and the total number of PSMs above the score threshold. PEAKS software employs the decoy fusion method and concatenate the decoy and target sequences of the same protein together as a “fused” sequence (21). Enzyme specificity was set as C-terminal to Arg and Lys, without allowing cleavage at proline bonds and a maximum of four missed cleavages. N-terminal pyroGlu, Met oxidation and Gln/Asn deamidation were set as variable modifications. No fixed modifications were set for the protein search. Tryptic digestion from rabbit phosphorylase B (Waters) was used as internal standard for label free quantification (2 pmol/μl) using a mass tolerance of 20 ppm, a retention time shift tolerance of 2 min, minimum 3 different peptides with a FDR set at 0.1%.

##### Bioinformatics

PSORTb version 3.0.2 was used for the prediction of protein cellular compartment (http://www.psort.org/psortb/) (22). Further refinement was performed for lipoprotein annotation that were sorted from unknown identifications using the precompiled genome annotation by DOLOP (http://www.mrc-lmb.cam.ac.uk/genomes/dolop/) (23).

##### Flow Cytometry

*E. coli* strains were grown for 16 h in liquid culture. Bacteria were then pelleted and washed with d-PBS at 8000 × *g* for 5 min. Bacteria were then blocked with d-PBS containing 3% (w/v) BSA for 15 min and incubated with mouse anti-OMV serum diluted in d-PBS with 1% (w/v) BSA (1:500) for 1 h. After washes, samples were incubated with Alexa Fluor 488 goat anti-mouse IgG (1:500) (Molecular Probes) for 30 min. Finally, bacteria were fixed with 3.7% (w/v) formaldehyde for 20 min and flow cytometry analysis was performed using FACS Canto II flow cytometer (BD Biosciences, San Jose, CA).

##### Recombinant Antigen Production

The gene fragment encoding BrkA, corresponding to protein residues 43–726, was amplified by PCR from *B. pertussis* W28 9K/129G genomic DNA. The PCR fragment was cloned into the pET15-TEV vector, a modified version of the pET15 vector (Novagen, Merck, Darmstadt, Germany), constructed to express N-terminal His-tagged (TEV cleavable) proteins by replacing the multiple cloning site of pET15 with a His-TEV-ccdB-chloramphenicol cassette amplified from the SpeedET vector (24). Protein expression was performed in *E. coli* BL21 (DE3) cells, by using EnPresso B growth systems (BioSilta, Oulu, Finland) supplemented with 100 μg/ml ampicillin. Bacteria were grown at 30 °C for 12 h, and recombinant protein expression was then induced by the addition of 1 mm isopropyl β-d-1-thiogalactopyranoside (IPTG) at 25 °C for additional 24 h. Proteins were extracted from the insoluble fraction with 6 m of guanidinium chloride and then purified by immobilized metal ion affinity chromatography (IMAC) using HiTRAP in 8 m Urea, 100 mm NaH_2_PO_4_ (pH 8) 10 mm Tris HCl (pH 8), 500 mm imidazole (GE Healthcare Life Sciences) and refolded by multistep dialysis in 50 mm NaH_2_PO_4_ (pH 7.5), 300 mm NaCl, 1% (v/v) glycerol.

##### Mouse Aerosol Challenge Model

BALB/c mice (10 female/group, 6-week old) (Charles River Laboratories International Inc.) received three intraperitoneal immunizations, with a 4 weeks interval, with aluminum hydroxide adjuvanted recombinant BrkA (10 μg per dose) or with adjuvant only. A bacterial suspension of BP536 from a 24-hour culture was generated at 2 × 10^9^ CFU/ml in d-PBS with 1% (w/v) casein salts (Sigma). BrkA-vaccinated and control mice were placed in a closed chamber in which the bacterial suspension was administered as an aerosol for 10 min using a nebulizer device with an administration rate of 0.5 ml/min. *B. pertussis* infection was monitored by performing colony forming units (CFU) counts on lungs from groups of 10 mice 7 days after aerosol challenge. Lungs were aseptically removed and homogenized in 2 ml of sterile physiological saline buffer with 1% (w/v) casein salts on ice. Ten microliters of undiluted homogenate or of serially diluted homogenates were spotted in triplicate onto Bordet-Gengou agar plates, and the number of CFU was estimated after 3 days of incubation at 37 °C. Statistical analysis was performed using unpaired *t* test (Mann-Whitney test).

## RESULTS

### 

#### 

##### OMV Purification, Visualization, Enumeration and Sizing

**Fig. 1.**
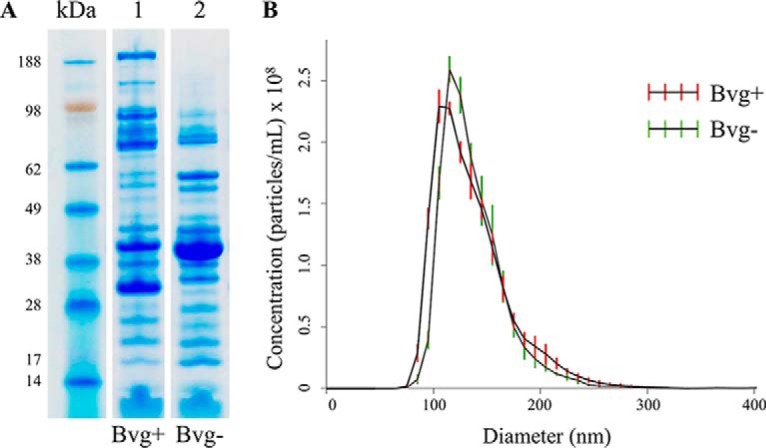
**OMV from Bvg+ and Bvg− strains have distinct protein composition but similar size and total protein amount per vesicle.** *A*, SDS-PAGE and Coomassie blue staining of 10 μg (protein content) of OMV samples from *B. pertussis* BP536 Bvg+ (lane 1) and its Bvg− derivative BP537 (lane 2). *B*, Nanoparticle tracking analysis measurement of OMV preparation (1 mg protein/ml) showing the sizes and total number of OMV per ml.

To identify possible Bvg-regulated outer membrane adhesins, we compared the protein composition of OMV from two *B. pertussis* strains, the Tohama I-derivatives BP536 and BP537 representative of the Bvg+ and Bvg− phases, respectively. OMV were purified using a detergent-free method from cell-free culture supernatants through ultracentrifugation. Equal amounts with respect to total protein quantities from both preparations were loaded onto SDS-PAGE gel (Fig. 1*A*) showing significant differences between the two samples representing Bvg+ and Bvg− phases. Several high molecular weight protein bands appear to be exclusive to Bvg+ OMV, as well as two major bands with apparent molecular weight of 42 and 32 kDa, whereas one intense protein band of apparent molecular weight around 40 kDa seems to be majorly expressed in Bvg− OMV. The 40 kDa protein band was excised, destained and digested with trypsin for peptide mass fingerprinting (PMF) identification: outer membrane porin protein BP0840 was identified with a sequence coverage of 27% (supplemental Fig. S1). To evaluate the actual number and the size of vesicles we used nanoparticle tracking analysis (NTA), a method for direct, real-time visualization of nanoparticles in liquids through the tracking of the Brownian movement of individual particles, as previously reported (25). OMV samples from BP536 and BP537, normalized to the same protein concentration of 1 mg/ml, contained 6 × 10^11^/ml and 6.3 × 10^11^/ml OMV-particles, respectively. The size distribution of OMV isolated from both strains was in the 70–230 nm diameter range with the majority of the OMV particles at 115 nm (Fig. 1*B*). These results indicated that, despite the dramatic difference in protein composition of the two OMV samples, 1 mg of proteins from each OMV preparation as measured with the Lowry assay, corresponds to the same number of vesicles, around 6 × 10^11^ blebs.

##### Adhesion of B. pertussis OMV to Human Respiratory Cells and Inhibition of B. pertussis Adhesion with Anti-OMV Serum

**Fig. 2.**
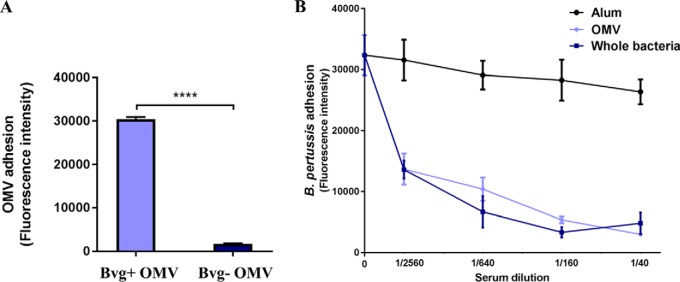
**Adhesiveness of *B. pertussis* OMV and impact of anti-OMV serum on *B. pertussis* adhesion to A549 cells:** *A*, Cells were incubated with OMV (10 ng/μl) for 6 h and, after extensive washes to remove unbound vesicles, cells were fixed and stained with mouse anti-OMV primary antibody and with AlexaFluor488-conjugated anti-mouse secondary antibody. Cell-associated OMV were quantified by fluorescence reading at Ex/Em 485/535 nm. Results represent mean ± S.D. of one representative of three independent experiments each performed in triplicates, *** = *p* < 0.001. *B*, Sera from 10 mice immunized with either OMV, whole bacteria or aluminum hydroxide as control were pooled, serially diluted in infection medium and incubated with labeled wild-type *B. pertussis* BP536 for 1 h. A549 cells were then infected with the bacteria/sera mixtures for 1 h and, after extensive washes to remove unbound bacteria, cell-associated bacteria were quantified by fluorescence reading at Ex/Em 485/535 nm. Results represent mean ± S.D. of one representative of three independent experiments each performed in triplicates.

To validate the employment of Bvg+ OMV as a source of adhesins to be selected as potential vaccine candidates, we checked whether Bvg+ OMV were enriched with adhesive molecules in comparison to Bvg− OMV. The two different OMV samples were added to seeded A549 respiratory epithelial cells and their ability to adhere was measured by immuno-fluorescence using mouse anti-OMV serum: we found that only Bvg+ OMV were able to bind to A549 cells (Fig. 2*A*). This result prompted us to determine whether antibodies raised against antigens present in Bvg+ OMV could have anti-adhesive properties on *B. pertussis*. We tested the ability of anti-OMV sera to inhibit adhesion of BP536 to A549 respiratory epithelial cells. Fluorescent bacteria were pre-incubated with mouse pooled sera in a range of four dilutions and A549 cells were infected as described previously (26). Mouse anti-sera were raised to Bvg+ OMV and whole bacteria, adsorbed to aluminum hydroxide and collected after the third immunization together with control sera from mice immunized with adjuvant only. We found that anti-OMV serum conferred a substantial reduction of adhering bacteria even at low serum concentrations. Interestingly, sera raised against whole bacteria, therefore targeting the entire membrane antigen repertoire, showed a comparable kinetic of inhibition. Anti-alum serum was included in the experiments as negative control and, as expected, did not induce any inhibition of *B. pertussis* adhesion (Fig. 2*B*).

##### Comparative Proteomic Analysis

**Fig. 3.**
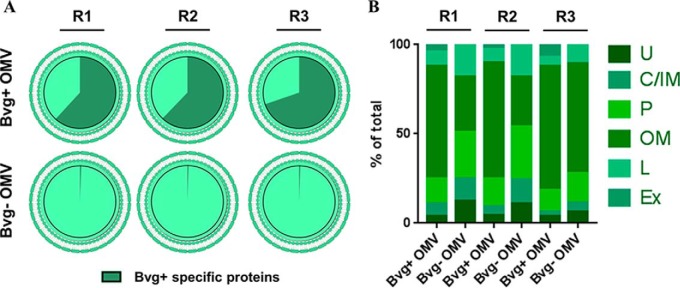
**Comparative proteomic analysis of *B. pertussis* OMV: protein quantification through LC-MS/MS and prediction of their subcellular localization.** *A*, Bvg+ specific proteins amount: Bvg+ specific proteins (green) were identified as proteins showing a 4-fold amount increase in Bvg+ *versus* Bvg− OMV and representing less than 0.5% of the total amount of Bvg− OMV. *B*, Distribution of quantified proteins in the distinct subcellular localizations based on prediction with PSORTb and DOLOP software: Outer Membrane (OM), Periplasm (P), Lipoprotein (L), Inner Membrane (IM), Cytoplasm (C), Extracellular (Ex), Unknown (U). R1 = biological replicate 1; R2 = biological replicate 2; R3 = biological replicate 3.

OMV samples from BP536 and BP537 were digested by trypsin after TCA precipitation. Proteomic analysis was performed by LC-MS/MS using a DDA approach. A total of 247, 155, and 372 proteins were quantified in the first, second and third biological replicate, respectively (supplemental Table S1). This variability was expected considering the high sensitivity of the Bvg regulon to slight environmental changes, as also proven in previous reports (27, 28). Therefore, each biological replicate was analyzed independently to identify the differentially expressed proteins in the Bvg+ *versus* Bvg− phase. Even if some proteins were identified exclusively in the Bvg+ phase, we set a threshold to determine the Bvg+ specificity: proteins showing a 4-fold increase in Bvg+ *versus* Bvg− phase and representing less than 0.5% of the total amount of proteins in Bvg− OMV were considered Bvg+ specific. Despite the mentioned differences in the absolute quantity of each protein in each biological replicate, ∼64% of the total protein amount in Bvg+ OMV appeared to be Bvg+ specific (Fig. 3*A*) and represented less than 0.6% of the total protein amount of Bvg− OMV. The localization of the quantified proteins was predicted according to PSORTb software and refined for lipoprotein annotation using DOLOP software. The analysis predicted ∼90 and ∼80% of the total protein amount to be either outer membrane proteins, lipoproteins, extracellular or periplasmic proteins, in Bvg+ and Bvg− OMV respectively and in all three biological replicates (Fig. 3*B*). Around 10% of the total protein amount had an unknown prediction of localization in both OMV samples but only a minority of proteins was predicted to be inner membrane or cytoplasmic, thus confirming that vesicles are generated by outer membrane blebbing and not by bacterial lysis.

##### Vaccine Antigen Selection

We applied three main criteria for antigen selection: putative antigens should be Bvg+ phase specific (*i.e.* showing at least a 4-fold increase as compared with Bvg−), they should have a predicted outer membrane localization according to PSORTb and they should represent at least 1% of the total amount of proteins present in Bvg+ OMV. These criteria must be true in at least 2 out of the three biological replicates. The list of the resulting selected antigens is reported in Table I. Excluding the proteins already part of the currently available aP formulation, we focused on six promising candidates to be assessed for their adhesive properties and vaccine potential: BrkA, Vag8, TcfA, SphB1, BipA, and BfrD. Interestingly, five of the selected antigens had a predicted autotransporter structure and they were identified with high amino acid sequence coverage in both the passenger domain and the β-barrel translocator domain, indicating that both domains are associated to OMV (supplemental Fig. S2).

**Table I:** OMV-based antigen selection. Antigens were selected from the total proteins quantified in OMV based on the following criteria: Bvg regulation, localization prediction, and abundance. Percentages refer to the total protein composition of OMV from either Bvg+ or Bvg− phase in the three biological replicates

Accession	Protein name [*Bordetella pertussis* Tohama I]	Replicate 1		Replicate 2		Replicate 3	
		% Bvg+	% Bvg−	% Bvg+	% Bvg−	% Bvg+	% Bvg−
NP_882013.1	BrkA autotransporter^ab^	12.96%	0.08%	10.69%	0.00%	19.21%	0.01%
NP_880571.1	FHA filamentous hemagglutinin/adhesin^ca^	17.95%	0.02%	15.33%	0.00%	1.43%	0.00%
NP_879839.1	69K pertactin autotransporter^ca^	5.44%	0.01%	11.48%	0.00%	10.68%	0.00%
NP_879974.1	TcfA tracheal colonization factor^ab^	4.26%	0.00%	3.28%	0.00%	6.95%	0.00%
NP_879893.1	BipA outer membrane ligand binding protein^ab^	1.56%	0.33%	5.27%	1.16%	9.06%	0.17%
NP_879104.1	SphB1 autotransporter subtilisin-like protease^ab^	6.28%	0.00%	6.71%	0.00%	0.48%	0.00%
NP_880953.1	Vag8 autotransporter^ab^	5.04%	0.01%	4.39%	0.00%	3.53%	0.00%
NP_879666.1	BfrD TonB-dependent receptor^b^	1.55%	0.02%	1.42%	0.00%	2.48%	0.00%

^a^ = known *B. pertussis* protective antigens.

^b^ = antigen selected for further analysis.

^c^ = antigen currently included in a commercial vaccine.

##### Recombinant E. coli Strains Adhesion and Adhesion Inhibition Assays

**Fig. 4.**
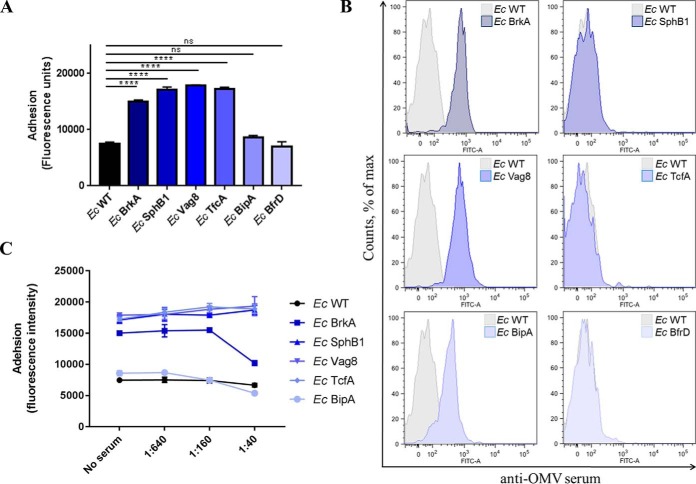
**Functional and immunological characterization of the selected antigens heterologously expressed on *E. coli*.** *A*, Adhesion of recombinant *E. coli* strains on A549 cells. A549 cells were infected with bacteria for 3 h and, after extensive washes to remove unbound bacteria, cells were fixed and stained with rabbit anti-*E. coli* primary antibody and with AlexaFluor488-conjugated anti-rabbit secondary antibody. Cell-associated bacteria were quantified by fluorescence reading at Ex/Em 485/535 nm. Results represent mean ± S.D. of one representative of three independent experiments each performed in triplicates, **** = *p* < 0.0001, ns = nonsignificant. *B*, FACS analysis on recombinant *E. coli* strain with anti-OMV serum: recombinant *E. coli* strains were collected in early exponential phase, stained with mouse anti-OMV primary antibody and with AlexaFluor488-conjugated anti-mouse secondary antibody, and finally fixed with 4% formaldehyde. Recognition of surface exposed *B. pertussis* antigens was checked by flow cytometry using FACS Canto II flow cytometer (BD Biosciences). *C*, Impact of anti-OMV serum on *E. coli* adhesion to A549 cells: sera from mice immunized with OMV (10 mice/group) were pooled, serially diluted in infection medium and incubated with recombinant *E. coli* strains for 1 h. A549 cells were then infected with the bacteria/sera mixtures for 3 h and, after extensive washes to remove unbound bacteria, cells were fixed and stained with rabbit anti-*E. coli* primary antibody and with AlexaFluor488-conjugated anti-rabbit secondary antibody. Cell-associated bacteria were quantified by fluorescence reading at Ex/Em 485/535 nm. Results represent mean ± S.D. of one representative of three independent experiments each performed in triplicates.

In order to evaluate the adhesive properties of each single protein, we exploited *E. coli* as an heterologous background system for surface exposure of the selected antigens. We generated *E. coli* strains constitutively expressing the selected full-length proteins and checked for their ability to bind to A549 respiratory epithelial cells as compared with wild type *E. coli*. A549 cells were infected with *E. coli* strains for three hours, then washed to remove unbound bacteria, fixed and stained with an anti-*E. coli* antibody. We found that BrkA, SphB1, Vag8 and TcfA conferred adhesive ability to *E. coli* when expressed on the membrane. On the contrary, heterologous expression of BipA and BfrD had no effect on strain adhesiveness (Fig. 4*A*). We continued the analysis by testing if the *E. coli* strains singularly expressing the selected antigens were detected with the anti-OMV serum we had generated. By flow cytometry, anti-OMV serum was able to recognize the specific heterologously expressed BrkA, Vag8 and BipA (Fig. 4*B*). This result showed that not only BrkA, Vag8 and BipA antibodies had been elicited after mice immunization with *B. pertussis* Bvg+ OMV, but also that these proteins are properly expressed and surface-exposed on *E. coli*. Finally, to determine whether the inhibition of *B. pertussis* adhesion promoted by anti-OMV serum correlated with the presence of specific antibodies against the selected antigens we tested its inhibitory effect on the adhesion of recombinant *E. coli* strains. We found that anti-OMV serum caused reduction of adhering *E. coli* at high serum concentration only when BrkA was expressed on the bacterial surface. Unexpectedly, Vag8-expressing *E. coli* was not inhibited despite the presence of specific anti-Vag8 antibodies in the serum (Fig. 4*C*).

##### Recombinant BrkA Protein Production and Vaccination

**Fig. 5.**
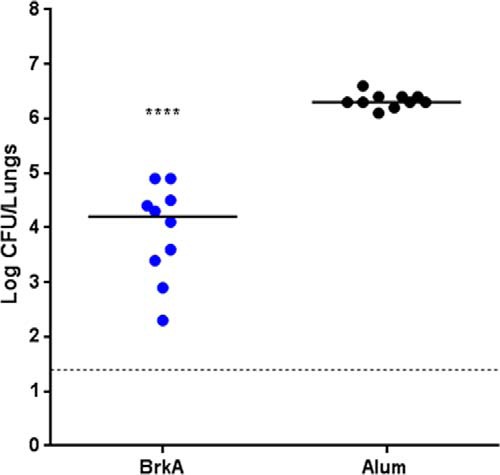
**Protection induced by BrkA in the mouse *B. pertussis* aerosol challenge model:** *B. pertussis* infection was followed by performing CFU counts on lungs from groups of 10 mice 7 days after aerosol challenge. Protection is reported as compared with mice immunized with the adjuvant only. Results represent mean ± S.D.; **** = *p* < 0.0001.

To analyze the protective potential of BrkA, recombinant His-tagged protein was expressed, purified and used to immunize mice three times, 4 weeks apart. Fifteen days after the final vaccination, mice were challenged with aerosolized *B. pertussis* and 7 days later the bacterial load in the lungs was evaluated by CFU counting. This experiment showed that immunization with recombinant BrkA resulted in about 100 fold reduction of bacterial load in the lung as compared with control mice treated with adjuvant only (Fig. 5).

## DISCUSSION

The increase in pertussis outbreaks and the recent observations with the employment of the baboon model for pertussis infection clearly indicated that a more potent vaccine is needed to prevent not only the disease but also the colonization and therefore transmission of the bacterium. The control of the bacterial burden is governed by multiple mechanisms leading to bacterial killing and interference to the infection process. Among them, the presence of functional antibodies at the epithelial barrier able to target key adhesins is a valuable countermeasure to inhibit the attachment of bacteria and initiation of colonization. Aiming at preventing *B. pertussis* colonization of the human respiratory tract, we checked the ability of *B. pertussis* OMV to bind to lung A549 cells and the ability of anti-OMV serum to inhibit *B. pertussis* adhesion to the same cell line. Interestingly, Bvg+ OMV, but not Bvg− OMV, were able to adhere to human respiratory cells, thus confirming their enrichment with Bvg-activated adhesins. Moreover, anti-OMV sera proved to be particularly powerful in preventing *B. pertussis* adhesion to respiratory epithelial cells. Clearly, the whole inhibitory effect on bacterial adhesion is likely determined by the additive effects of different antibodies targeting various antigens displayed by OMV and present on *B. pertussis* surface. We therefore decided to deepen the analysis at the single protein level, looking for proteins present in Bvg+ OMV that could elicit anti-adhesive antibodies after immunization. The comparative proteomic analysis between OMV from Bvg+ and Bvg− allowed the identification of hundreds of proteins and showed that ∼64% of the total protein amount in Bvg+ OMV are specific for that phase and their amount drops to ∼0.6% in Bvg− OMV. This finding agrees with the well-established regulation of the virulence genes by the BvgAS two-component system in *B. pertussis*. Interestingly, almost the totality of the Bvg+ specific proteins were predicted to be outer membrane proteins and among them we found the best known and characterized *B. pertussis* adhesins such as FHA, 69K and Fimbriae. Also, Pertussis Toxin was identified as Bvg+ specific whereas Adenylate Cyclase Toxin and Dermonecrotic Toxin were not identified at all. On the other hand, there was a general increase in the amount of all the non Bvg+ specific proteins in Bvg− OMV and it is tempting to hypothesize that most of the proteins in Bvg− OMV are overrepresented to compensate for the lack of the Bvg+ outer membrane proteins. The outer membrane porin protein BP0840 perfectly supported this hypothesis, resulting to be the most abundant protein in Bvg− OMV in all the biological replicates. The NTA analysis further supported the hypothesis of a compensative mechanism, showing no differences in size distribution and quantity of proteins per vesicle in both OMV samples. The protein composition of OMV described in the present study differs substantially from what has been shown in a previous report (28). Although the high quantity of BrkA and Vag8 is confirmed in both studies, the overall composition in terms of presence and relative abundance is drastically different. This is likely because of the distinct OMV purification methods and *B. pertussis* strains employed in the two studies. The proteomic analysis presented in this study allowed the identification of an initial list of putative antigens, which were narrowed down to 8 interesting candidates. This antigen selection resulted in seven known *B. pertussis* protective antigens including two of the current aP antigens, thus validating our strategy to identify virulence factors. It is intriguing to underline that proteomic analysis of two currently circulating *B. pertussis* strains under *in vitro* Bvg+ and Bvg− conditions (29), combined to *in silico* prediction for surface- expression, resulted in a panel of top 15 candidates which perfectly mirrors the OMV-based antigen selection described in our study. Then, albeit with variations in the strains and methods employed, the membrane composition of OMV seems to closely reflect the composition of the bacterial outer membrane. We decided to focus our attention on the most abundant Bvg+ outer membrane proteins and interestingly five autotransporter proteins were at the top of our list. Autotransporters are possibly the simplest bacterial secretion systems (type V): they consist only of a single polypeptide chain organized in a passenger and translocator domain and they can autonomously translocate across the outer membrane (30). Therefore, the heterologous expression of the autotransporters on the surface of *E. coli* was selected to characterize their adhesive properties. This approach allowed the demonstration that BrkA, TcfA, SphB1 and Vag8 conferred to *E. coli* a significant increased ability to adhere to lung epithelial cells. To the best of our knowledge, this is the first time that *B. pertussis* autotransporters were rationally characterized for their adhesive properties exploiting a heterologous *E. coli* background. The involvement in adhesion of BrkA was previously demonstrated in *in vitro* analysis and in mouse infection studies with *B. pertussis* mutant strains showing 2-fold and 2-log reduction in the ability to adhere to cell lines and to colonize the murine lungs, respectively (31, 32). Also, the loss of TcfA was previously shown to cause 10-fold reduction in the number of bacteria isolated from tracheas after *B. pertussis* aerosol challenge (33). Nevertheless, deletion of individual virulence factor genes could have limited effects on the ability of *B. pertussis* to efficiently infect the respiratory tract of mice, suggesting they may perform redundant functions. This was the case of a Vag8 deletion mutant, which was as efficient as the parental *B. pertussis* strain in colonizing murine lungs (34). On the contrary, our study showed that Vag8 is also involved in adhesion and not only in serum resistance as previously described (35). In other cases, the generation of single knock-outs could be misleading. In fact, SphB1 loss has an indirect effect on Filamentous Hemagglutinin which is not cleaved anymore from *B. pertussis* surface therefore rendering the mutant strain even more adhesive than the wild-type parental strain (36) but in this study, we showed that SphB1 itself can contribute to the overall adhesiveness of *B. pertussis*. Two of the heterologously expressed antigens, BipA and BfrD, did not confer an adhesive phenotype to *E. coli*. Although this result could be expected for BfrD, which is reported to be involved in iron acquisition (37), it was quite controversial for BipA, which is partially characterized in the literature as the major adhesin in the intermediate phase between Bvg+ and Bvg− (38). This virulence phase (Bvg(i)) was described to be involved in the transmission of *B. pertussis* from host to host and a BipA deletion mutant of a Bvg(i)-locked *B. pertussis* strain displayed a reduced ability to colonize the nasal cavity of mice compared with the parental strain (39). BipA was also identified as the most abundant surface-associated biofilm protein (40) therefore it is likely involved in bacterium-bacterium interactions as well as host-pathogen interactions. Our results were in contrast with the previous reports but, in the absence of specific antibodies, we could not rule out that BipA is not properly expressed and surface exposed on *E. coli*. We therefore took advantage of sera raised against OMV to explore whether the heterologously expressed antigens were recognized on the surface of *E. coli*. Only three proteins, BrkA, Vag8 and BipA, were specifically recognized on *E. coli* surface by anti-OMV serum, thus excluding the possibility that BipA was not properly surface-exposed on the outer membrane. Finally, we checked the ability of anti-OMV serum to inhibit recombinant *E. coli* adhesion to lung A549 cells. The inhibitory effect of anti-OMV serum was only observed on the adhesion of BrkA-expressing *E. coli*, demonstrating the functionality of anti-BrkA antibodies. Consistently with the fact that SphB1 and TcfA-expressing *E. coli* strains were not recognized by anti-OMV serum, no inhibition was observed on the adhesiveness of these strains. Unexpectedly, anti-OMV serum was not able to inhibit Vag8-expressing *E. coli* strain; whether this is because of low antibodies level or to weak antibody functionality needs further clarification. As far as BfrD is concerned, the protein did not result to have a contribution in adhesion nor to be recognized by anti-OMV serum on *E. coli* surface, therefore was excluded from the adhesion inhibition assay. Still, given its predicted structure of integral outer membrane protein, we cannot exclude that it is not properly expressed on the surface of *E. coli*. The FACS analysis using anti-OMV serum on *E. coli* strains also gave us an indirect result on the ability of the selected antigens to be immunogenic in the context of OMV. Immunization of mice with OMV results in a wide antibody repertoire somehow reflecting what happens during a natural infection with *B. pertussis* or during a vaccination with wP. Clearly, immunogenicity does not necessarily correlate with protein abundance and, in the complexity of the OMV lipid bilayer, some abundant proteins might not induce that high antibody response, whereas some less abundant proteins might be immuno-dominant and induce higher responses. Although the presence of anti-BrkA, anti-Vag8 and anti-BipA antibodies correlated with high abundance of these proteins on OMV, the absence of anti-SphB1 and anti-TcfA antibodies was unexpected given the high quantity of these proteins on vesicles. Nevertheless, immunization of mice with recombinant proteins could have a completely different outcome and elicit specific anti-adhesive antibodies, as previously demonstrated. Indeed, the protective role of autotransporters is a peculiar trait of *B. pertussis* and thus far, five autotransporters have been shown to confer protection in the mouse model (either aerosol or intranasal challenge) when expressed as recombinant proteins, including Prn (41), TcfA (42), SphB1 (29), Vag8 (29), and BipA (40) suggesting that *B. pertussis* evolved this class of proteins as virulence strategy to colonize the host. Here, we demonstrated that immunization with recombinant BrkA resulted in significant protection against lower respiratory tract infection of *B. pertussis* in the mouse aerosol challenge model. This was different with respect to what previously reported using the mouse intranasal challenge model, where BrkA resulted as an added value only when formulated with aP and not as stand-alone vaccine (43); however, the two studies differ both in the immunization route and in the bacterial infection administration. Finally, we contributed to unravel the mechanism of protection induced by anti-BrkA antibodies showing the inhibitory properties against the bacterial adhesion. In conclusion, BrkA proved to be a promising candidate antigen to improve existing aP vaccines for use in humans. Whether such multi-component aP vaccines that include BrkA and possibly further virulence factors of *B. pertussis* other than pertussis toxin, filamentous hemagglutinin, pertactin and fimbriae have superior efficacy over existing aP vaccines remains to be determined.

Taken together, these results suggest that spontaneously released OMV from *B. pertussis* provide a potential source of protective antigens able to specifically target the colonization step. The inclusion of BrkA and possibly further adhesins may provide a higher protection to current aP vaccines answering to the need of improving the impact of vaccination on the bacterial clearance.

## DATA AVAILABILITY

Raw mass spectrometry data were deposited in the publicly accessible repository MassIVE (Project number: MSV000081702; Proteome Exchange PXD008179).

## Supplementary Material

Supplemental Data
