# Supplementary material for: Outer Membrane Vesicles (OMV)-based and Proteomics-driven Antigen Selection Identifies Novel Factors Contributing to Bordetella pertussis Adhesion to Epithelial Cells
Source: Mol Cell Proteomics. 2017 Dec 4;17(2):205–15. doi: 10.1074/mcp.RA117.000045 (PMC5795387; doi:10.1074/mcp.RA117.000045)
Supplement: Supplemental Data [file supp_17_2_205__index.html]

Outer Membrane Vesicles (OMV)-based and Proteomics-driven Antigen Selection Identifies Novel Factors Contributing to Bordetella pertussis Adhesion to Epithelial Cells — Proteomic Study of B. pertussis OMV Detects Novel Antigens — Supplemental Data 

# Outer Membrane Vesicles (OMV)-based and Proteomics-driven Antigen Selection Identifies Novel Factors Contributing to *Bordetella pertussis* Adhesion to Epithelial Cells

## Supplemental Data

undefined

- Supplementary materials and figures - Supplementary materials and figures
- Supplementary Table 1 - Table S1. Comparative proteomics data: proteins quantified in OMV from BP536 and BP537 strains
