# Supplementary material for: Outer Membrane Vesicles (OMV)-based and Proteomics-driven Antigen Selection Identifies Novel Factors Contributing to Bordetella pertussis Adhesion to Epithelial Cells
Source: Mol Cell Proteomics. 2017 Dec 4;17(2):205–15. doi: 10.1074/mcp.RA117.000045 (PMC5795387; doi:10.1074/mcp.RA117.000045)
Supplement: Supplemental Data [file supp_RA117.000045_4590_1_supp_23794_p06wpc.pdf]

APPENDIXES

Supplemental Materials and Methods

In Gel Protein Digestion and MALDI-TOF Mass Spectrometry Analysis

Protein bands were excised from the gels, washed with 50 mm ammonium bicarbonate (Fluka Chemie AG), MS-grade acetonitrile (Sigma) (1:1, v/v), washed once with pure acetonitrile, and air-dried. Dried spots were digested for 18 h at 37 °C in 20 µl of 5 mm ammonium bicarbonate and 12 ng/µl sequencing grade modified trypsin (Promega). After digestion, 0.6 µl were spotted onto a matrix PAC target (Prespotted AnchorChip 96, set for proteomics, Bruker Daltonics) and air-dried. Spots were washed with 0.6 µl of 70% ethanol, 0.1% trifluoroacetic acid. Mass spectra were acquired on an Ultraflex MALDI-TOF/TOF mass spectrometer (Bruker Daltonics) in reflectron, positive mode in the mass range of 700–3500 m/z. Spectra were externally calibrated by using a combination of standards prespotted on the target. MS spectra were analyzed with FlexAnalysis (version 2.4, Bruker Daltonics) using default parameters and manually revised. Protein identification was performed using MASCOT search engine (version 2.2.1) by searching in *B. pertussis* Tohama I NCBI protein database (3,425 protein entries, release date: 2001/11/06). Search parameters were for peptide mass fingerprint analysis were: enzyme, trypsin; missed cleavage, 1; peptide tolerance, 100 ppm; variable modifications, methionine and tryptophane oxidation; mass values, [M+H]+ ions and monoisotopic.

Table S1. Comparative proteomics data: proteins quantified in OMV from BP536 and BP537 strains.

With LC-MS/MS analysis, 389 proteins were identified and quantified in three biological replicates of Bvg+ (BP536) and Bvg- (BP537) OMV. For each protein, accession number, proteomic data and protein annotation from B. pertussis Tohama I database are listed. In addition, the average of the coverage (n=3 technical replicates), number of detected peptides and peak area with standard deviation are retrieved from PEAKS version 8. Subsequently, theoretical molecular weight (MW) is given with the calculated percentage for each protein in the total sample as described in Materials and Methods. Finally, the fold increase of each protein amount in Bvg+ vs. Bvg- is reported with the PSORTb and DOLOP prediction of subcellular localization.

FIG. S1. Peptide mass fingerprinting (PMF). OMV from Bvg+ (lane 1) and Bvg– (lane 2) *B. pertussis* strains were resolved by SDS-PAGE. The most abundant protein band (highlighted in red) was excised, destained and digested with trypsin for PMF identification. Outer membrane porin protein BP0840 was identified with sequence coverage of 27%. MALDI spectrum was reported. For each identified peptide the following information were reported: start-end amino acid position of the identified peptide within the protein sequence; experimental *m/z*; observed *m/z*; calculated mass; difference (error) between the experimental and calculated mass (delta); number of missed cleavage sites; Mowse score from MASCOT; expectation value for the peptide match; MASCOT rank of the peptide match; uniqueness of peptide sequence in the database; amino acid peptide sequence, list on *m/z* loaded during the search.

FIG. S2. Autotransporters sequence coverage of identification. Sequence coverage of the five autotransporters selected as interesting vaccine candidates: percentage and graphical representation as obtained with Peaks 8 software analyzing the third biological replicate as a representative of the three experiments.

Figure S1

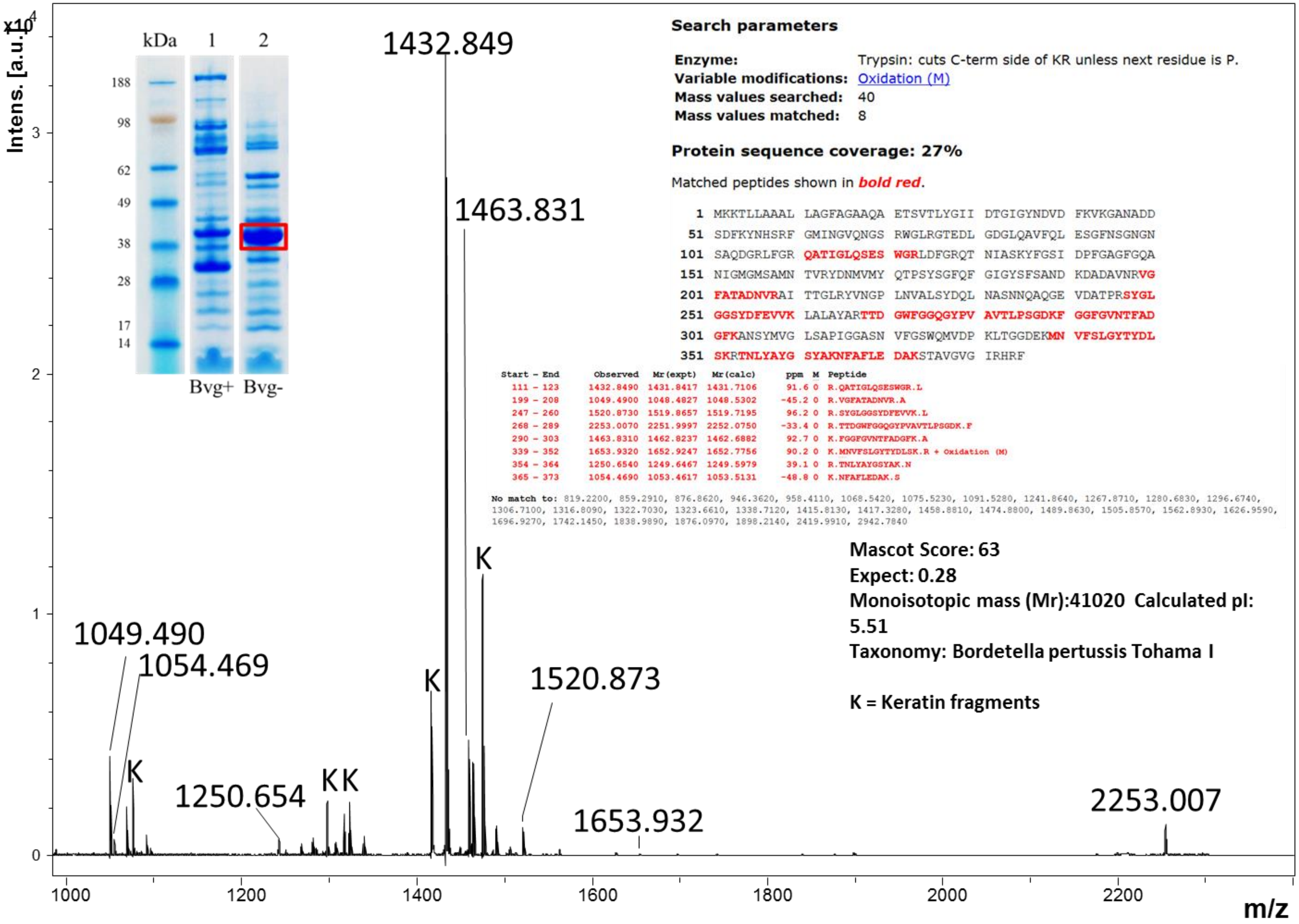
x10^4
Intens. [a.u.]
kDa 1 2
188
98
62
49
38
28
17
14
Bvg+ Bvg-
1432.849
1463.831
1049.490
1054.469
1250.654
1520.873
1653.932
2253.007
K
K K
m/z
Search parameters
Enzyme: Trypsin: cuts C-term side of KR unless next residue is P.
Variable modifications: Oxidation (M)
Mass values searched: 40
Mass values matched: 8
Protein sequence coverage: 27%
Matched peptides shown in bold red.
1 MKKTLLAAAL LAGFAGAAQA ETSVTLYGII DTGIGYNDVD FKVKGANADD
51 SDFKYNHSRF GMINGVQNGS RWGLRGTEDL GDGLQAVFQL ESGFNSGNGN
101 SAQDGRLFGR QATIGLQSES WGRLDFGRQT NIASKYFGSI DPFGAGFGQA
151 NIGMGMSAMN TVRYDNMVMY QTPSYSGFQF GIGYSFSAND KDADAVNRVG
201 FATADNVRAI TTGLRYVNGP LNVALSYDQL NASNNQAQGE VDATPRSYGL
251 GGSYDFEVVK LALAYARTTD GWFGGQGYPV AVTLPSGDKF GGFGVNTFAD
301 GFKANSYMVG LSAPIGGASN VFGSWQMVDP KLTGGDEKMN VFSLGYTYDL
351 SKRTNLYAYG SYAKNFAFLE DAKSTAVGVG IRHRF
Start - End Observed Mr(expt) Mr(calc) ppm M Peptide
111 - 123 1432.8490 1431.8417 1431.7106 91.6 0 R.QATIGLQSESWGR.L
199 - 208 1049.4900 1048.4827 1048.5302 -45.2 0 R.VGFATADNVR.A
247 - 260 1520.8730 1519.8657 1519.7195 96.2 0 R.SYGLGGSYDFEVVK.L
268 - 289 2253.0070 2251.9997 2252.0750 -33.4 0 R.TTDGWFGGQGYPVAVTLPSGDK.F
290 - 303 1463.8310 1462.8237 1462.6882 92.7 0 K.FGGFGVNTFADGFK.A
339 - 352 1653.9320 1652.9247 1652.7756 90.2 0 K.MNVFSLGYTYDLSK.R + Oxidation (M)
354 - 364 1250.6540 1249.6467 1249.5979 39.1 0 R.TNLYAYGSYAK.N
365 - 373 1054.4690 1053.4617 1053.5131 -48.8 0 K.NFAFLEDAK.S
No match to: 819.2200, 859.2910, 876.8620, 946.3620, 958.4110, 1068.5420, 1075.5230, 1091.5280, 1241.8640, 1267.8710, 1280.6830, 1296.6740, 1306.7100, 1316.8090, 1322.7030, 1323.6610, 1338.7120, 1415.8130, 1417.3280, 1458.8810, 1474.8800, 1489.8630, 1505.8570, 1562.8930, 1626.9590, 1696.9270, 1742.1450, 1838.9890, 1876.0970, 1898.2140, 2419.9910, 2942.7840
Mascot Score: 63
Expect: 0.28
Monoisotopic mass (Mr):41020 Calculated pI: 5.51
Taxonomy: Bordetella pertussis Tohama I
K = Keratin fragments

Figure S2

Accession	Protein name [Bordetella pertussis Tohama I]	Sequence coverage	%
NP_882013.1	**BrkA** autotransporter	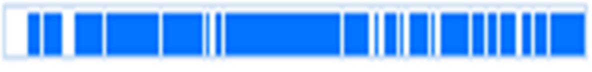	85
NP_879104.1	**SphB1** autotransporter subtilisin-like protease	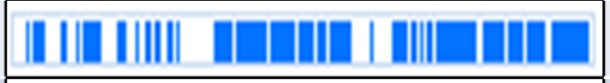	69
NP_879893.1	**BipA** outer membrane ligand binding protein	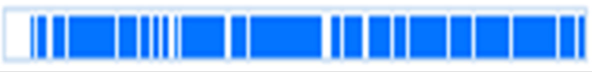	81
NP_880953.1	**Vag8** autotransporter	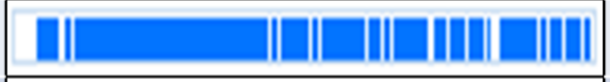	87
NP_879974.1	**TcfA** tracheal colonization factor	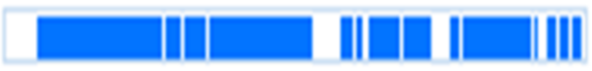	82
